# The longitudinal dispositions of people diagnosed with adjustment or severe stress disorders

**DOI:** 10.1186/s12888-024-05904-y

**Published:** 2024-06-18

**Authors:** Daniel Poremski, Jayaraman Hariram, Wei Kang Wong, Pui Wai EU, Cheng Lee

**Affiliations:** 1 Institute of Mental Health, 10 Buangkok View, Singapore, 539747 Singapore; 2 Yong Loo Lin School of Medicine, The National University of Singapore, Singapore, 117597 Singapore

**Keywords:** Adjustment disorder, Acute stress disorder, Administrative data, Longitudinal disposition

## Abstract

**Background:**

Adjustment and stress-related disorders are prevalent among psychiatric service users. Despite their prevalence, little is known about their prognosis. To reduce that gap, the present article documents the service use and diagnostic outcomes of people with adjustment or stress-related disorders presenting at Singapore’s largest psychiatric emergency department.

**Methods:**

Administrative data from 2014 to 2021 was retrieved to follow a group of 683 service users whose first-ever psychiatric presentation in 2014 warranted a diagnosis of adjustment or stress-related disorder. People were grouped a priori depending on whether different diagnoses were recorded within 7 days, 9 months, after 9 months or not at all. Survival curves characterized conversion to other diagnoses and engagement with healthcare services. Service use outcomes include the number of hospitalizations, outpatient appointments, emergency department visits, and prescriptions.

**Results:**

Sixty-one percent (*n* = 417) never received another diagnosis over the 8-year period. This group used emergency services most and received the most pharmacotherapy shortly after their first visit. Of those who received another diagnosis, depression, personality disorders, and psychotic disorders were the most common. Those who received another diagnosis within 7 days (*n* = 70, 10%) received it on their first day of hospitalization (IQR 1–1), making the most use of inpatient services. The group who received another diagnosis within 9 months (*n* = 105, 15%) did so after 42 days (IQR 26–84) and had the highest relative number of deaths. Those who received another diagnosis after 9 months (*n* = 91, 13%) did so after 1,134 days (IQR 613–1,823) and had the longest period of engagement but made the least use of any psychiatric service, potentially suggesting a group whose early index diagnosis heralded vulnerability to future disorders.

**Conclusions:**

A large group of service users with acute stress or adjustment disorders will likely never be given another psychiatric diagnosis and appear to disengage following an initial period of high-intensity service use. The group that received a different diagnosis after the 9-month mark had prolonged contact with services but low intensity of service use and may represent a target for preventative intervention to help them improve their stress-managing skills and avoid developing other disorders.

**Supplementary Information:**

The online version contains supplementary material available at 10.1186/s12888-024-05904-y.

## Background

Adjustment and stress-related disorders are some of the most frequent diagnoses made at emergency services in Singapore [1], and at outpatient services in general [2, 3]. Despite their prevalence, they are under-studied and frequently subsumed under the general category of stress disorders along with posttraumatic stress disorder, which often receives the bulk of the attention [4]. Given the public health implications of this knowledge gap, several have called, decade after decade, for increased focus on their etiology, prognosis, and treatment [5–9].

The diagnosis of an adjustment disorder is, by definition, limited to six months post the emergence of the stressor, and one month in the case of acute stress disorders [2]. After these time points, other diagnoses may be more appropriate if symptoms persist. Much of what is known about illness progression can be traced to seminal early studies, which indicated that, after a 5-year follow-up of 100 patients, 71% of adults and 44% of adolescents with adjustment disorder were well [5]. The adult group developed major depressive disorder and alcohol abuse disorders. In contrast, adolescents developed a wider range of psychiatric disorders, including schizophrenia, bipolar disorder, antisocial personality disorder, drug abuse, and major depressive disorders [5]. With fewer recent epidemiological studies that replicate these early findings, it is difficult to know if people with the diagnosis eventually develop other conditions or have long-term mental health needs [4], and systematic reviews of available evidence continue to emphasize the importance of studying the course of the disorders [8]. Recent studies have identified that in some subpopulations, symptoms may increase over time, marking a trajectory toward a more severe disorder. In a study by O’ Donnell et al. (2019), trauma survivors who had adjustment disorder three months after exposure were 2.67 times more likely to meet criteria for a more severe psychiatric disorder (including PTSD, major depressive disorder, and generalized anxiety disorder) at 12 months. Further, in this same study, 34.6% of those with adjustment disorder at three months still met the diagnostic criteria at twelve months, suggesting persistent symptoms.

Although emerging evidence indicates that stress-related disorders may be a gateway to more severe psychiatric disorders, it is essential to highlight that adjustment disorder is associated with significant adverse outcomes in and of itself. Consultant liaison psychiatry research indicates adjustment disorder is significantly associated with suicidality and self-harm, at similar proportions to depressive disorders [4, 10]. Inpatient populations have likewise been found to have high rates of self-harm and suicidality in adjustment disorder cases compared to other diagnoses. It has also been a comorbid diagnosis for people recovering from cancer or other chronic and severe physical illnesses [8].

Treatments are diverse, but guidelines are under-developed [11, 12]. While it ranked 7th of all psychiatric categories (44 categories were provided for ranking) in day-to-day practice amongst surveyed psychiatrists, it ranked in the lowest percentile (33% portion) of ease of use or goodness of fit [3]. This indicates that clinicians did not feel comfortable with the official diagnostic definitions or clinical criteria when diagnosing clients. These uncertainties correspond to the weaknesses and failures of the current disorder definitions in ICD-10 or DSM-IV/-V. Because of these difficulties, the use of these diagnoses has been at the center of debate concerning their importance and merit [13, 14].

### Aim

The current project sought to observe the changes in diagnoses that occurred on the medical records of people initially given an adjustment disorder or stress-related diagnosis at the Institute of Mental Health in Singapore. Subsequent goals were to determine if these groups used emergency services differently throughout their illness, used outpatient services differently, or had different hospitalizations. Finally, the study sought to determine if pharmacological interventions provided to these groups differed in any respect.

## Methods

This study employed retrospective administrative data to generate a timeline lengthy enough to address the research aims. No primary data was collected for this article. Administrative data was used to identify people who engaged with the host institute’s emergency services and received a diagnosis of adjustment disorder or severe stress reaction (Online supplement, Table A, note PTSD was excluded) over the index year 2014. Previous work on the institute’s emergency service attendees quantified this population as 2,927 distinct service seekers, or 18% of all emergency service visitors over 2014 [1]. Those diagnosed over the index year had their administrative records searched retrospectively to 2012 to determine the date of first contact and if the identified index diagnosis was the first on record. Two years was chosen, as previous research on this administrative dataset showed that, even amongst frequent users, very few have service use patterns that extend beyond 2 years in retrospect [15]. Of the 2,927 service users previously recorded as having attended the hospital’s emergency services with eligible diagnoses in 2014, only those with no previous record were retained for the present article. This ensured that our sample was most likely treatment and diagnosis naïve at the time of the index diagnosis. Those given an eligible diagnosis but with a record of another pre-existing psychiatric diagnosis were excluded. The decision to exclude people with a previous psychiatric diagnosis related to the wish to generate conclusions about cases for which the disorder of interest represented the only psychiatric condition for which they would have been treated. This exclusion also reduces the confounding previous psychiatric history may have on psychiatric service use outcomes. With the exclusion, it is reasonable to attribute treatments given and outcomes observed to the diagnosis of interest. All diagnoses assigned from 2014 onwards, including PTSD, were considered and dated to determine which diagnoses were eventually warranted. Service use data, including additional emergency service visits, outpatient visits, and admissions, were also retrieved to explore the long-term service demand of this population (to December 2021). All-cause mortality was recorded in the administrative dataset, but due to the nature of the death registry in Singapore, it was not possible to determine, for the purposes of this project, the cause of death or if a psychiatric diagnosis was warranted at the time of death. No unstructured case notes were available to the research team for the purpose of the project.

The Institute of Mental Health’s Institutional Research Review Committee (reference #807–2022), as well as the national ethics committee, National Health Group Domain Specific Review Board, Domain F1 (#2022/00684) approved the study and granted a waiver of consent because the study team collected anonymous administrative data that had already been generated for clinical purposes.

### Setting

Singapore is a small, highly urbanized, densely populated equatorial nation-state in Southeast Asia. It has a population of approximately 5.6 million inhabitants of Chinese, Malay, and Indian descent. It has obligatory national service for men but was not actively deployed in any conflict over the study period. It consistently ranks low on Crime Indexes, with the main types of crimes recorded being outrage of modesty (indecent exposure), voyeurism, shop theft, theft in dwelling, and rioting [16].

The host institute represents the main and largest source of psychiatric care in Singapore. Its catchment area extends to the entire country. It has 800 acute and 1200 chronic care beds divided into four geographic zones. Because it encompasses the entirety of Singapore, most cases with psychiatric concerns are routed, at some point of their illness trajectory, to the hospital and recorded in its medical records. It is also national policy for forensic services and uniformed agencies to route all psychiatric emergencies to its emergency services, as it is the only institute in the nation with appropriate facilities for such service users [17]. So, while the dataset is derived from one institute, this institute serves Singapore in its entirety.

Most of the psychiatrists practicing in Singapore were trained locally. The local training system follows the US model and is accredited by the Accreditation Council for Graduate Medical Education—International [18]. While discussions about diagnostic criteria often follow the DSM nomenclature [19], the medical records record diagnoses as SNOMED and ICD-10 codes. Throughout 2014, the emergency services were served by a team of consultant or senior consultant psychiatrists during office hours and by Senior Residents after hours (all completed the MRC Psych Examination with a minimal 3 years of post-graduate experience in psychiatry).

### Dataset

The dataset contained every psychiatric interaction between service users and the host institute, the date of every interaction, prescriptions, diagnoses, sex, ethnicity, dates of birth and death, emergency service referral source and visit disposition. Psychiatric interactions were grouped into emergency services (visits for psychiatric emergencies exclusively), inpatient services (includes only hospitalizations for psychiatric concerns, including those for observation, further investigation or involuntary detention under the Mental Health Act), outpatient services (includes only services offered by psychiatrists) and pharmaceutical services (includes predominantly psychotropics, but also medications for side effects and metabolic comorbidities, further details given below). While the pharmaceutical services group features in all prior groups, it was treated independently because prescriptions could span the transition between settings and stretch over multiple months.

Referral sources were only examined for emergency service visits, as inpatient referrals came almost exclusively via emergency services per local policy, and outpatient service referrals almost exclusively came from internal referrals for discharged service users (either via hospital discharges or emergency service visit discharges). Referrals sources were grouped into self-referrals (where service users present of their own volition or with the support of family members), other healthcare providers (grouping all other hospitals, general practitioners, and healthcare providers), legal authorities (includes all service users compelled to present to services by police, the courts or other correctional services), and uniformed agencies (includes all forms of military personnel, national servicemen, or armed civil servicemen who would be compelled to attend emergency services by their organization or superior officer). Length of hospitalization was calculated as the difference between the time of admission to the hospital and the time of discharge from the hospital. The length of engagement with services was the difference between the date of the index visit and the date of the last recorded interaction (either emergency visit, outpatient visit, or inpatient discharge). Correspondingly, disengagement was the last point of contact recorded for the service user.

The variable documenting interaction disposition includes categories for discharge ( where a service user leaves the hospital without plans to return), hospitalization (where a service user requires admission to the institute’s wards), transfer to other health services (where a service user is discharged to the care of another hospital for the treatment of a physical illness, or nursing home) follow-up appointments ( where the service user leaves the hospital with a plan for their return either at outpatient services or inpatient services), and finally other dispositions (where a service user is discharged into the care of correctional services or abscond (rarely used). No category captures information about loss to follow-up or recovery. Therefore, the research team cannot conclude whether the absence of visits represents recovery or service avoidance. However, given the nationwide nature of the institute, it is unlikely that people requiring psychiatric care disengage entirely with the host institute in pursuit of care elsewhere.

### Pharmaceutical services data

The list of medications was extensive. To facilitate the reporting of medications, only those responsible for the top 70% of the volume of prescriptions were considered. The 70% threshold was chosen as it reasonably distinguished between medications routinely given and those which were infrequently given and did not affect the inclusion of psychotropics. The 70% contained 30 of approximately 300 distinct medications. Censoring the list to 30 rather than 300 simplified their categorization for subsequent analyses. Of these, only medications given for psychiatric conditions were kept, and general medicines not given for psychiatric disorders were dropped. This led to the exclusion of vitamins, supplements, insulin, pain medications, blood pressure medication and medications given for constipation. The list of medications is found in the online appendix.

### Analyses

A Kaplan–Meier survival curve was fitted to characterize the length of time between the first diagnosis of adjustment disorder and the emergence of other disorders. This characterized the number of people who continued to be diagnosed with adjustment disorders or stress-related disorders and how many were subsequently given other diagnoses. The cohort was then split along clinically relevant watersheds to group the sample a priori into four: 1) those that received a different diagnosis within a week. This time horizon was chosen because hospitalized service users would have, by this time, been reviewed by a second psychiatrist and have any update to their diagnosis recorded in their medical record. Service users who might be discharged but continued to be in distress warranting additional services would have returned to the hospital’s emergency service or been given an emergency outpatient appointment within that 7-day window. 2) those who received a different diagnosis within 9 months of the original diagnosis. This time horizon was chosen because the DSM-V diagnostic criterion indicates that an adjustment disorder should be diagnosed within 3 months of the stressor and not last more than 6 months. If issues persist, a different diagnosis should be given. 3) those who received a different diagnosis after 9 months. And 4) those who did not receive a different diagnosis at all over the 8-year period.

A second Kaplan–Meier survival analysis was conducted to determine the difference between the groups in terms of their engagement with mental health services, with their last recorded date of engagement with the hospital as the failure condition. The ordered nature of the group was specified in the log-rank test for equality of survivor functions. Cox hazard ratios were calculated to supplement the Kaplan–Meier survival curve and quantify the adjusted relationships between the groups and the service use outcome. The adjusted Cox model included age and referral source as variables that differed significantly between the groups. Neither sex nor ethnicity was significantly different between the groups and, consequently neither was included in the adjusted model.

Descriptive statistics were used to characterize each group’s pattern of service use and demographic variables. ANOVA and student’s t-test were used to determine the significance of the associations between the groups and categories, and continuous variables respectively. For significant associations between groups and continuous outcomes, post hoc Bonferroni multiple-comparison tests were conducted. Significant differences are noted by indicating which two groups differed with an indication of the direction of the difference. Chi-squared tests were used to determine if the categorical variables differed by groups. Fisher’s exact test was used where the chi-squared test cells were smaller than five. Given that many continuous outcomes were heavily skewed, non-parametric tests replaced the ANOVA. Specifically, Kruskal–Wallis equality-of-populations rank tests were conducted to test for differences between outcomes with skewed data.

Statistical analyses are conducted in STATA 16 and Python 3.11, with significance set at two-tailed 0.05.

## Results

The sample contained 683 individuals (whose diagnosis given over the index year represents their first on record). Clinical wisdom indicates that the predominant stressors included financial, occupational, and interpersonal stressors. Stressors related to war, civil unrest, political conflict, natural disasters, assaults, motor vehicle accidents, or chronic illness were unlikely to be seen in this population. However, individual-level clinical data was not reviewed to document the precise nature of the stressors systematically.

Table 1 presents the four groups and their demographic characteristics. Sixty-one percent of the sample (417/683) never received another diagnosis over the course of the 8 years of administrative data. Of the remaining, 10.2% (70/683) received another diagnosis within 7 days of their first. Fifteen percent (105/683) received another diagnosis before 9 months, and 13.3% (91/683) received another diagnosis after 9 months (Table 1).

**Table 1:** Group demographics and diagnoses

	1	2	3	4	Test of significance
Convert within	< 7 days	< 9 months^a^	> 9 months	Never	
n (%)	70 (10.2%)	105 (15.4%)	91 (13.3%)	417 (61.1%)	
Male (%)	35 (9.7%)	60 (16.6%)	54 (14.9%)	213 (58.8%)	Chi-squared *p* = 0.298
Age at time of index diagnosis (mean, 95%CI)	35.2, 31.6–38.9	34.1, 31.2–37.1	31.1, 27.8–34.4	30.8, 29.6–32.1	ANOVA *p* = 0.011, group 1 > 4
Ethnicity					Chi-squared *p* = 0.060
Chinese	45(9.7%)	81(17.5%)	62(13.4%)	274(59.31%)	
Malay	11(15.3%)	9(12.5%)	12(16.7%)	40(55.6%)	
Indian	7(8.4%)	5(6.0%)	14(16.9%)	57(68.7%)	
Other	4(6.0%)	11(16.7%)	3(4.5%)	48(72.7%)	
Days to conversion (median, IQR)	1, 1–1	42, 26–84	1134, 613–1823	33, 25–44^b^	Kruskal–Wallis equality-of-populations rank test *p* < 0.0001
Diagnosis					Not calculated
PTSD	3 (50.0%)	2 (33.3%)	1 (16.7%)		
Depressive disorders	20 (25.0%)	38 (47.5%)	22 (27.5%)		
Psychotic disorders	10 (37.0%)	8 (29.6%)	9 (33.3%)		
Personality disorders	4(21.0%)	8 (42.1%)	7 (36.9%)		
Alcohol use	3 (23.1%)	5 (38.5%)	5 (38.5%)		
Anxiety disorders	3 (27.3%)	2 (18.2%)	6 (54.5%)		
Bipolar disorder	0 (%)	3 (100%)	0 (%)		
Childhood disorders	1 (10.0%)	6 (60.0%)	3 (30.0%)		
Conduct disorder	1 (25.0%)	2 (50.0%)	1 (25.0%)		
Drug use	2 (22.2%)	3 (33.3%)	4 (44.4%)		
Learning disability	1 (33.3%)	2 (66.7%)	0 (%)		
Obsessive compulsive disorders	3 (37.5%)	2 (25.0%)	3 (37.5%)		
Opioid	2 (25.0%)	2 (25.0%)	4 (50.0%)		
Sleep disorders	1 (33.3%)	1 (33.3%)	1 (33.3%)		
Other^c^	16 (24.6%)	21 (34.4%)	25 (41.0%)		

^a^Groups are mutually exclusive

^b^Of the 51 with a formal note that “no mental illness” was recorded at the time of their follow-up visit

^c^Other disorders included Epilepsy, Impulse control disorders, somatization disorders, gambling disorders, neurotic disorders, transitional situational disturbance, disruptive behaviours, and intentional self-harm

**Fig. 1:**
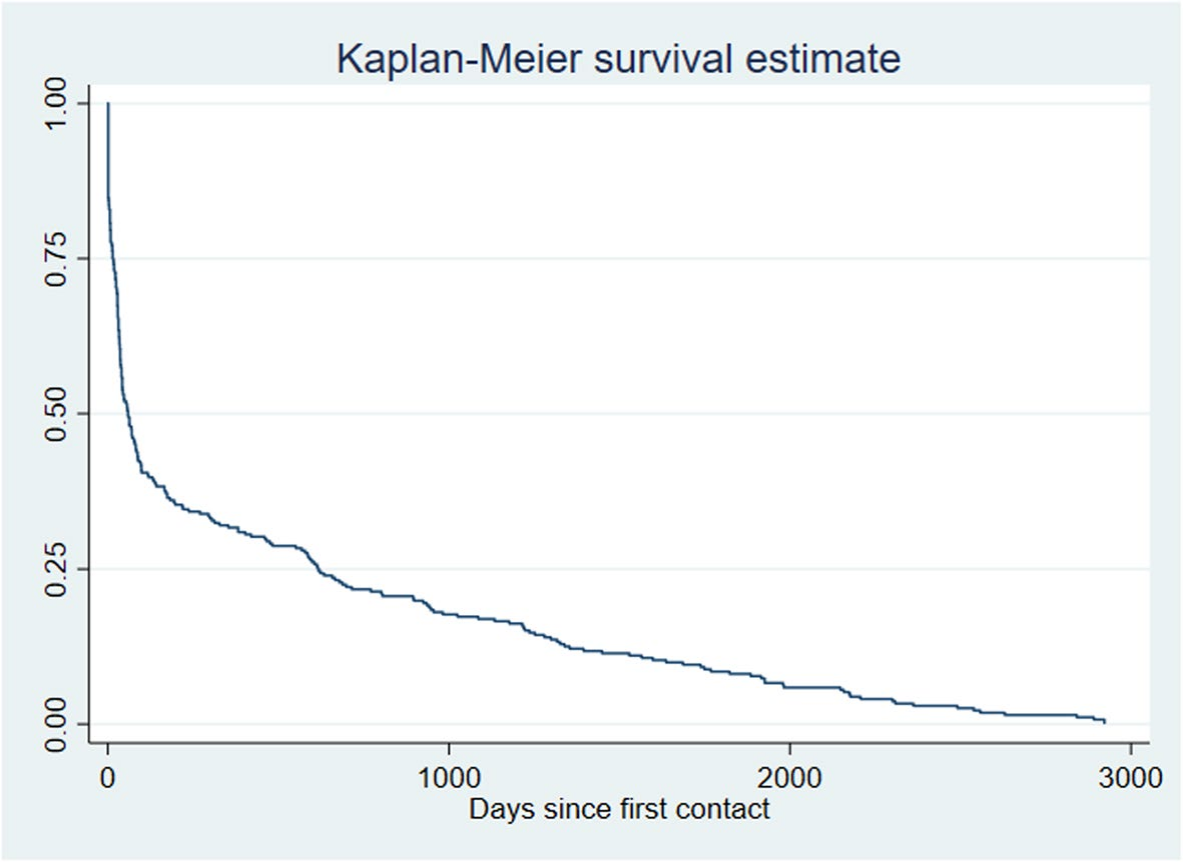
Kaplan Meier survival curve based on the time between receiving a diagnosis of adjustment disorder or severe stress reaction and receiving any other psychiatric diagnosis

Fifty-one service users with an eligible index diagnosis were explicitly given a tag of “no mental illness” at a dated follow-up encounter, explaining why a value could be calculated for conversion. The survival curve in Fig. 1 illustrates the sample’s transition to other diagnoses.

**Fig. 2:**
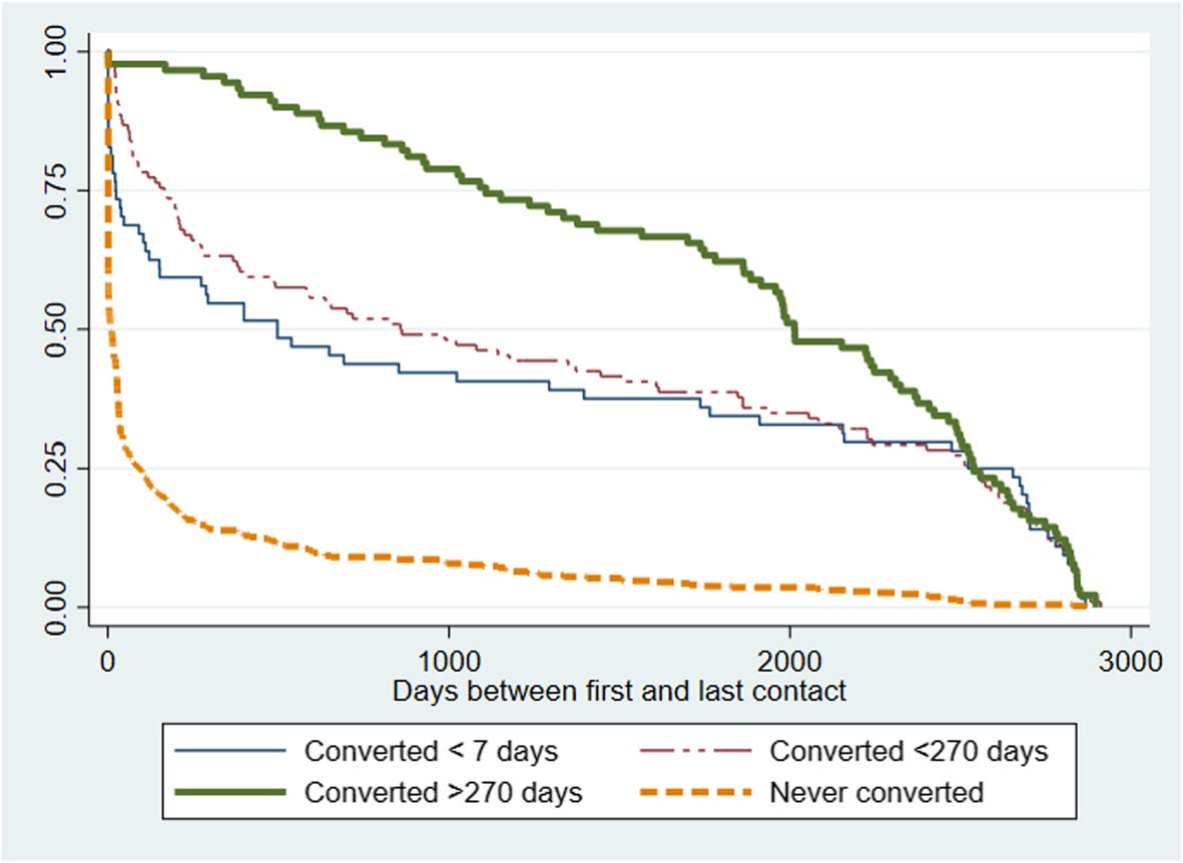
Kaplan Meier survival curve based on the time between first and last recorded engagement with mental health services

The groups, as can be expected, differed in terms of their engagement with hospital services. The first group that received a different diagnosis within 7 days had a median engagement with services of 497 days. The second group that received a different diagnosis within 9 months had a median engagement period of 858 days. The third group that converted after 9 months had the longest period of engagement, lasting a median of 2,013 days. The group that never received another diagnosis disengaged with services after a median of 10 days and never contacted any type of psychiatric services offered by the host institute thereafter. Figure 2 presents the survival curves for each group with the time elapsed between their first and last recorded visits with the hospital used to calculate the curves. The observation period maximum was 2,922 days or 8 years. Details are located in Table 2. Log-rank test for equality of survivor functions were significant (Χ^2^ (3) = 164.14, *p* < 0.0001). Cox proportional hazards model, adjusted for age and referral source with the point at which service users disengaged with services as the failure, hazard ratios are given in Table 2. Hazard ratios confirmed the group that never received another diagnosis had a higher risk of disengaging with services compared to the group that converted within 7 days, with a hazard ratio of 2.8 (95%CI 2.07–3.64). The group that converted after 9 months had a hazard ratio of 0.7 (95%CI 0.52–0.99), suggesting that service users from this group had longer engagement than those who converted within 7 days.

**Table 2:** Service use rates and characteristics

	1	2	3	4	Test of significance
Convert within	< 7 days	< 9 months^a^	> 9 months	Never	
Size (%)	70 (10.2%)	105 (15.4%)	91 (13.3%)	417 (61.1%)	
Median (IQR) days of engagement with services	497 (30- 2552)	858 (166–2544)	2013 (1091–2558	10 (1–107)	Kruskal–Wallis equality-of-populations rank test *p* < 0.0001
Days of observation (time at risk)	71,514	131,861	167,062	88,155	
Cox hazard ratio (standard error, p)^b^	1	0.9506 (0.1526, *p* = 0.753)	0.7184 (0.1209, *p* = 0.049)	2.7745 (0.3964, *p* < 0.0001)	
Proportion receiving psychiatric medications^c^	77.1%	93.3%	83.5%	37.7%	Chi-squared *p* < 0.0001
Medication prescriptions^d^	6,389	12,744	7,831	2,232	
Portion hospitalized once or more	77.1%	60.0%	65.9%	40.5%	Chi-squared *p* < 0.0001
Distinct hospitalizations	210 (23.5%)	263 (29.4%)	186 (20.8%)	236 (26.4%)	
hospitalizations /10,000 days	29.4	19.9	11.1	26.8	Chi-squared *p* = 0.011
Length of hospitalization in days (mean, 95% CI)	9.9, 7.5–12.2	19.1, 9.5–28.6	12.4, 7.8- 17.0	5.8, 4.3–7.4	Kruskal–Wallis equality-of-populations rank test *p* < 0.0001
Portion attending at least one outpatient appointment	77.1%	93.3%	80.2%	41.5%	Chi-squared *p* < 0.0001
Outpatient visits	1019 (25.6%)	1398 (35.2%)	1021 (25.6%)	543 (13.6%)	
Outpatient visits /10,000 days	142.5	106.0	6.1	61.6	Chi-squared *p* < 0.0001
Portion attending only the index emergency visit	41.4%	29.5%	11.0%	77.5%	Chi-squared *p* < 0.0001
Emergency visits	353 (17.0%)	671 (32.3%)	402 (19.4%)	649 (31.3%)	
Emergency visits /10,000days	49.4	50.9	24.1	73.6	Chi-squared *p* < 0.0001
Emergency visit disposition					Chi-squared *p* < 0.0001
Hospitalizations	188 (23.4%)	245 (30.5%)	145 (18.0%)	226 (28.1%)	
Transfers to other health service	16 (29.1%)	17 (30.9%)	6 (10.9%)	16 (29.1%)	
Discharges	11 (9.1%)	27 (22.3%)	27 (22.3%)	56 (46.3%)	
Follow up appointment	132 (14.2%)	355 (38.1%)	187 (20.1%)	258 (27.7%)	
Other disposition	6 ( 3.7%)	27 (16.6%)	37 (22.7%)	93 (57.1%)	
Referral source for emergency services					Chi-squared *p* < 0.0001
Uniformed Agencies	11 (6.6%)	29 ( 17.5%)	35 (21.1%)	91 (54.8%)	
Other Healthcare provider	83 (20.7%)	155 (38.6%)	56 (13.9%)	108 (26.9%)	
Legal Authority	66 (11.3%)	122 (20.9%)	117 (20.0%)	279 (47.8%)	
Self	193 (20.9%)	365 (39.5%)	194 (21.0%)	171 (18.5%)	
Deaths	2 (8.7%)	8 (34.8%)	4 (17.4%)	9 (39.1%)	Fisher’s exact test *p* = 0.120
Mean age at death (min–max)	50.2 (42.2–58.2)	49.6 (31.7–62.4)	71.1 (48.9–100.3)	63.5 (42.3–95.7)	Not calculated

^a^Groups are mutually exclusive

^b^Cox model adjusted for referral source and age. Sex and Ethnicity had no statistically significant effect on the hazards model

^c^Fig. 3 illustrates the rate of prescription

^d^Psychiatric medications responsible for the top 70% of the volume are listed in the online appendix

The first group that converted within 7 days made the most use of outpatient and inpatient services, with the highest admissions rate following their emergency service visits. They also had the lowest engagement with legal authorities or uniformed agencies. While the distribution of disorders to which service users converted appears somewhat uniform between the groups, this first group that converted within 7 days had lower levels of depression, personality disorder, and alcohol use disorders compared with the group that converted within 9 months. Psychosis-related disorders emerged in all groups but might be most prominent in this first group. Length of hospitalization also appears shorter in this group, but not significantly so. For this first group, the close temporal proximity between stressors, first engagement with psychiatric services, and subsequent diagnosis of a severe mental illness may suggest that the stressor played a role in the precipitation of the severe mental illness.

The second group, which converted within 9 months, made moderate use of all services compared to the other groups. This second group has the highest representation amongst self-referrals and referrals made by other hospitals. It also has a disproportionately excessive number of deaths, on average occurring at a relatively younger age.

The third group that converted beyond 9 months made the least use of services, with sharply less use of outpatient services, emergency services and admissions, despite having lengths of hospitalization similar to the other groups. It is possible that this third group had sparse contact with hospital services but eventually developed another psychiatric disorder near the end of the 8-year observation period. This is supported by the distribution of prescriptions, which peak around their conversion date and near the end of the observation period.

**Fig. 3:**
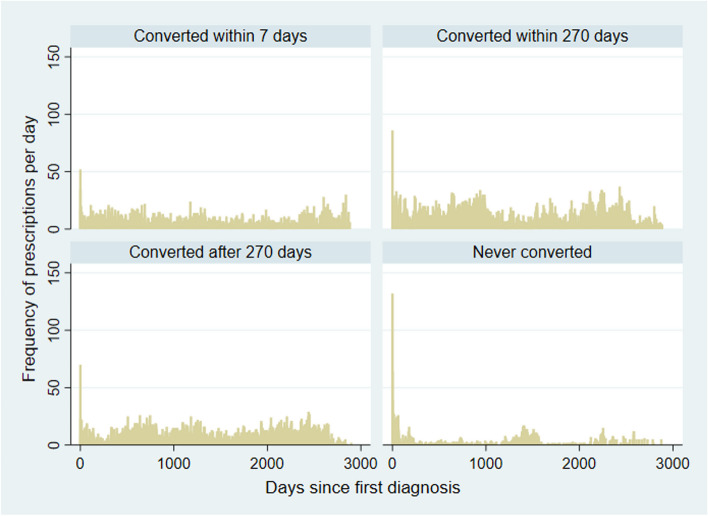
Number of prescriptions issued on each day since originally receiving a diagnosis of adjustment disorder or severe stress disorder

The fourth group that never converted had the greatest involvement with legal authorities and uniformed agencies, implying many involuntary visits. They were the group with the highest rate of discharge following their emergency service visits. When they were hospitalized, they had significantly shorter hospitalizations. Despite these lower rates of service use, they made the most frequent use of emergency services of all the other groups over their brief period of engagement with services. This likely represents a high intensity of service need, followed by long, if not total, disengagement with services. The fourth group also received the greatest number of prescriptions shortly after their first contact (Fig. 3).

Of the 23 deaths recorded, 9 (40%) had a severe stress reaction diagnosis at their last point of contact with services. Other diagnoses recorded in the records of the deceased group include depression (*n* = 3, 13%), psychotic disorders (*n* = 2, 9%), and other organic symptoms (*n* = 2, 9%). Three had no formal diagnosis at their last point of contact with the hospital. Anxiety disorders, drug use disorders, personality disorders and learning disabilities were recorded once each.

## Discussion

Adjustment disorders and acute stress reactions, despite their prevalence, represent one of the least understood and one of the least user-friendly diagnostic groups [3]. This incongruence represents a significant target for improvement as this group may be under-served despite the risk of harm borne by such service users [20]. The present study sought to add to the existing body of literature by examining the service use trajectories of service users diagnosed with an adjustment or severe stress disorder at the Institute of Mental Health’s emergency services.

Of the 2,927 emergency service visitors with a diagnosis of adjustment disorder [1], 683 had no prior history of contact with the hospital. The majority of this group (61.1%) never received another diagnosis at subsequent visits. While it is not possible, given the administrative data at our disposal, to confirm that no illness remained after this group’s disengagement with mental health services or that no disorder was diagnosed elsewhere, 12% of the group no longer met the criteria for a psychiatric illness at their last point of contact. Furthermore, as the largest source of psychiatric tertiary care in Singapore, it is unlikely that this group chose another source of psychiatric care which was able to serve them over the 8-year period without requiring further contact from the hospital.

Of the groups that eventually met the criteria for another psychiatric illness, the majority received a depressive disorder diagnosis, a personality disorder diagnosis, or a psychosis-related diagnosis. Anxiety and PTSD were not detected as frequently. This finding deviated slightly from previous research, noting that PTSD, major depressive disorder and generalized anxiety disorder may be the most common diagnoses detected in a cohort that had adjustment disorder (2019), though O’Donnell et al. focused on survivors of trauma, a fundamentally different population.

Our groups were structured to reflect a priori watershed moments in the illness trajectories [2], but the median time to conversion for each group suggests that the time horizon for receiving another diagnosis is quite different. We had expected the 9-month cleavage to be accompanied by proximal changes. Instead, the group that converted within 9 months on median converted just over a month after their original diagnosis (Table 1), in line with what is expected of severe stress reactions, though very few cases of PTSD were recorded. The group that converted beyond 9 months on median converted just shy of three years, possibly indicating an entirely new set of circumstances that warrant psychiatric care. This initial diagnosis of adjustment or severe stress disorder followed by a long gap (three years in our case) in service use may potentially herald a vulnerability to psychiatric illness. Neither the 6- nor 9-month mark appears to hold significance to the emergence of further psychiatric disorder.

Our prescription records showed that, all other things being equal, people who received more pharmacotherapy early appear to belong to a group that never developed another psychiatric diagnosis (Fig. 3). Because the development of symptoms that fit with other disorders occurs after the initial diagnosis and prescription, it is possible to suggest that the early intensive pharmacological treatment played a role in avoiding the development of conditions observed in the other groups. This fits with the proposed prophylactic effect of anxiolytics in the prevention of PTSD [21, 22]. Prophylactic administration of psychotropic medications to disrupt the physiological and psychological effects of stress hormones to prevent the development of PTSD has been contentious [21] though beta-blockers have a direct theoretical mode of action [22]. In this observational study, the prescription of anxiolytic and sedative-hypnotics appears to be higher in the group that never developed a subsequent psychiatric disorder. Additionally, this group that never developed another psychiatric diagnosis did not differ from the others in terms of the distribution of eligible index diagnoses, suggesting that the psychiatrists assessing the presenting complaint did not differentiate these service users from others with adjustment disorders or severe stress reactions at the point of assessment, but prescribed more medications to this group, nonetheless. Further research may contribute significantly to the health of the population by expanding the understanding of the relationship between stressors and the development of psychiatric illness and the role of pharmacological and cognitive behavioural interventions [23]. The prescription of propranolol, however, does not fit with this stress-modulating effect. Prescription records also show a cluster of activity approximately six years after the index year. This represents the period during which Singapore was most affected by the pandemic and its quarantine policy, which demonstrably affected emergency service use [24].

Deaths appeared to occur disproportionately in the group which converted within 9 months, at a younger age. Diagnoses related to severe stress reactions appear disproportionately represented amongst the group, which may fit with previous literature. Without adequate access to services, this group may experience a worsening of health, expressed in longer hospital admissions. In contrast, early detection of other psychiatric concerns and appropriately intensive treatment may reduce the possibility of deterioration [8]. While it is not possible to firmly identify the cause of death in these cases, the fact that the average age at the time of death was relatively low despite the group having the highest average age suggests premature deaths outnumber those in line with the Singapore life expectancy. Suicide and self-harm are known to affect people with adjustment disorders at a ratio like those seen in populations with depression [4, 10].

### Limitations

While the study has a long period of observation, the use of administrative data has its disadvantages [25]. The research team did not influence how service users were monitored, and their diagnosis remained entirely up to the discretion of their psychiatrists. This possibly introduces variability and bias in how cases were followed up and diagnosed. Additionally, the reliance on administrative data limits the research team’s ability to characterize the nature of the stressors leading to the index disorder. Without being able to characterize the nature of the stressors objectively, it may be difficult to generalize the study’s conclusions to other populations and locations beyond those which highly resemble Singapore’s densely urbanized, economically prosperous nature. Results cannot be generalized to locations where war, conflict or natural disasters are sources of stress and trauma. Additionally, the administrative data dictated which variables were available for analyses, making it impossible to control for confounders unless they were measured and recorded in the records.

It is also not possible to directly compare these results with those of studies that focused on people who experienced trauma [26] or with those that report the development of physical conditions that result from the index disorder [8]. Future research may benefit from documenting and classifying the exact nature of the stressor to determine if the nature of the stressors is associated with similar trajectories as those observed in the present study. Developing a robust predictive model would allow clinicians to intervene more confidently with prophylactic pharmacological interventions.

An additional limitation concerns the sample size of the index group. While it is large enough to support statistical comparisons, limiting the sample to only service users with no previous recorded history of mental illness may have led to the exclusion of most service users with acute stress disorders or adjustment disorders. However, the purpose of the study was to, as much as possible, follow people who were treatment-naïve and whose adjustment disorder diagnosis represented their first contact with psychiatric services. Extending the cohort to multiple years may have increased the sample size but would have reduced the observation period. Prioritizing the observation period better achieved the research objectives and reduced the possibility of subsequent service needs going undetected. Further research could shed light on the interaction between previous mental illness, acute stressors, and the manifestation of subsequent symptoms, though it is already well-accepted that people with mental illness are more frequently the target of violence [27].

Finally, the decision to group stress-related disorders with adjustment disorders may be questioned given the differing time criteria noted between both the DSM and ICD. However, both groups are similar because they depend e on the presence of an acute stressor and should have a time-limited duration. As a result, it is not uncommon to group these disorders [4]. Those wishing to generalize the findings of this study must keep in mind that clinicians may differ in their comfort diagnosing these disorders [3], potentially leading to different judgements about which is most applicable in any given case. Further analyses may be justified to treat stress-related disorders in isolation.

## Conclusion

The majority of people who presented to emergency services with symptoms that warranted a diagnosis of adjustment disorder or severe stress disorder appeared to develop no subsequent psychiatric illness. This group disengaged with services on median after 10 days but made the most use of emergency services. Despite being clinically similar to the groups that would eventually warrant another diagnosis, the group that never developed another psychiatric disorder received the most medication of any group early in their timeline. Of the 39% of people who do develop another condition, depressive, psychotic and personality disorders are the most common. Deaths appear to occur disproportionately in excess in this group, especially in the group that received an alternate diagnosis within 9 months of their first contact with mental health services.

At the time of diagnosis, the research sample was treatment- and diagnosis-naïve, and because they were all given stress or adjustment-related diagnoses, it is probable that they presented quite similarly to emergency services. However, these service users eventually experienced vastly different illness trajectories, and developing a better understanding of the nuanced differences between the groups, if detectable at the first point of contact, may help prescribe targeted interventions.

### Supplementary Information

Supplementary Material 1.

## Data Availability

The datasets generated and/or analysed during the current study are not publicly available because they were extracted from a narrow band of medical records and contain data that, when taken in their entirety and paired with other datasets, can be used to re-identify service users. The dataset supporting the conclusions of this article is available from the corresponding author upon reasonable request, subject to national and institutional ethics approval.
