# Supplementary material for: The longitudinal dispositions of people diagnosed with adjustment or severe stress disorders
Source: BMC Psychiatry. 2024 Jun 18;24:457. doi: 10.1186/s12888-024-05904-y (PMC11186233; doi:10.1186/s12888-024-05904-y)
Supplement: Supplementary file 1 — Supplementary Material 1. [file 12888_2024_5904_MOESM1_ESM.docx]

Table s1. ICD10 codes which qualify a service user as an index case

| ICD10 code | ICD10 diagnosis | n |
| --- | --- | --- |
| F430 | Acute stress reaction | 489 |
| F4320 | Adjustment disorder, unspecified | 90 |
| F4321 | Adjustment disorder with depressed mood | 18 |
| F4322 | Adjustment disorder with anxiety | 1 |
| F4329 | Adjustment disorder with other symptoms | 1 |
| F438 | Other reactions to severe stress | 55 |
| F439 | Reaction to severe stress, unspecified | 26 |
| Z600 | Problems of adjustment to life-cycle transitions | 3 |

Table s2. Medication distribution. Below the observed count cell is the corresponding expected count based on the null hypothesis, and off which the test of significance is based. Comparing the two illustrates which group is higher or lower than what is expected if there is no difference in prescribing practices between the groups. Chi Squared<0.001

| Medication | within 7 days | within 9 months | after 9 months | never |
| --- | --- | --- | --- | --- |
|  |  |  |  |  |
| Lorazepam | 839 | 1,926 | 658 | 370 |
| Expected | 830.0 | 1,655.6 | 1,017.4 | 290 |
| Hydroxyzine | 475 | 1,044 | 840 | 488 |
| Expected | 623.0 | 1,242.7 | 763.6 | 217.6 |
| Diazepam | 243 | 356 | 356 | 116 |
| Expected | 234.4 | 467.5 | 287.3 | 81.9 |
| Zopiclone | 253 | 58 | 133 | 5 |
| Expected | 98.3 | 196.0 | 120.4 | 34.3 |
|  |  |  |  |  |
| Fluvoxamine | 445 | 1,676 | 500 | 214 |
| Expected | 620.4 | 1,237.5 | 760.4 | 216.7 |
| Fluoxetine | 795 | 412 | 1,111 | 52 |
| Expected | 518.6 | 1,034.5 | 635.7 | 181.2 |
| Mirtazapine | 352 | 806 | 214 | 94 |
| Expected | 320.8 | 639.9 | 393.2 | 112.1 |
| Escitalopram | 202 | 419 | 418 | 59 |
| Expected | 240.3 | 479.3 | 294.5 | 83.9 |
| Sulpiride | 173 | 30 | 127 | 11 |
| Expected | 74.6 | 148.8 | 91.5 | 26.1 |
| Sertraline | 31 | 127 | 125 | 15 |
| Expected | 65.2 | 130.1 | 79.9 | 22.8 |
| Trazodone | 40 | 40 | 166 | 15 |
| Expected | 57.1 | 113.9 | 70.0 | 20 |
|  |  |  |  |  |
| Quetiapine | 572 | 1,601 | 1,395 | 38 |
| Expected | 789.1 | 1,574.0 | 967.2 | 275.7 |
| Olanzapine | 1,136 | 1,400 | 341 | 176 |
| Expected | 668.1 | 1,332.6 | 818.9 | 233.4 |
| Risperidone | 136 | 430 | 292 | 256 |
| Expected | 243.8 | 486.3 | 298.8 | 85.2 |
| Haloperidol | 151 | 264 | 82 | 134 |
| Expected | 138.1 | 275.4 | 169.2 | 48.2 |
| Clozapine | 21 | 207 | 211 | 0 |
| Expected | 96.1 | 191.6 | 117.7 | 33.6 |
|  |  |  |  |  |
| Propranolol | 182 | 396 | 355 | 11 |
| Expected | 206.6 | 412.1 | 253.2 | 72.2 |
|  |  |  |  |  |
| Sodium Valproate | 303 | 1,088 | 507 | 178 |
| Expected | 454.3 | 906.2 | 556.8 | 158.7 |
| Lamotrigine | 0 | 334 | 0 | 0 |
| Expected | 73.1 | 145.8 | 89.6 | 25.5 |
| Lithium | 40 | 130 | 0 | 0 |
| Expected | 37.2 | 74.2 | 45.6 | 13 |
|  |  |  |  |  |
|  |  |  |  |  |
| Total | 6,389 | 12,744 | 7,831 | 2,232 |
|  | 6,389.00 | 12,744.00 | 7,831.00 | 2,232.00 |
